# Age, Initial Central Retinal Thickness, and OCT Biomarkers Have an Influence on the Outcome of Diabetic Macular Edema Treated With Ranibizumab– Tri-center 12-Month Treat-and-Extend Study

**DOI:** 10.3389/fmed.2021.668107

**Published:** 2021-05-03

**Authors:** Chun-Ting Lai, Yi-Ting Hsieh, Chun-Ju Lin, Jia-Kang Wang, Chih-Ying Lin, Ning-Yi Hsia, Henry Bair, Huan-Sheng Chen, Chiung-Yi Chiu, Shao-Wei Weng

**Affiliations:** ^1^Department of Ophthalmology, China Medical University Hospital, China Medical University, Taichung, Taiwan; ^2^Department of Ophthalmology, National Taiwan University Hospital, Taipei, Taiwan; ^3^School of Medicine, College of Medicine, China Medical University, Taichung, Taiwan; ^4^Department of Optometry, Asia University, Taichung, Taiwan; ^5^Department of Ophthalmology, Far Eastern Memorial Hospital, Taipei, Taiwan; ^6^Department of Electrical Engineering, Yuan Ze University, Taoyuan City, Taiwan; ^7^An-Shin Dialysis Center, NephroCare Ltd., Fresenius Medical Care, Taichung, Taiwan

**Keywords:** age, central retinal thickness, diabetic macular edema, OCT biomarkers, ranibizumab, treat-and-extend regimen 5

## Abstract

**Objective:** We report the tri-center 1-year outcomes of a treat-and-extend (T&E) regimen in four-week intervals with ranibizumab for diabetic macular edema (DME).

**Methods:** In this retrospective study, all eyes received 3 monthly loading injections of 0.5 mg ranibizumab, followed by a T&E regimen for DME. Regression models were used to evaluate the associating factors for visual and anatomical outcomes.

**Results:** Ninety one eyes from 64 patients were enrolled. Mean LogMAR best-corrected visual acuity (BCVA) improved from 0.58 at baseline to 0.36 at month 12 and mean central retinal thickness (CRT) decreased from 411 μm at baseline to 290 μm at month 12. Younger age and eyes having thinner baseline CRT, with ellipsoid zone disruption (EZD), and without epiretinal membrane (ERM) were associated with better final CRT. Moreover, eyes with thicker baseline CRT tend to receive more injections. Among the parameters, only having ERM or EZD was associated with significant BCVA recovery.

**Conclusions:** A T&E regimen with ranibizumab by 4-week intervals is effective in improving BCVA and reducing CRT with efficacy notable starting from the third month. Clinical parameters including age, initial CRT, and presence of ERM or EZD significantly influenced therapeutic outcomes. Moreover, the presence of ERM should not preclude DME patients from receiving anti-VEGF therapy. Future studies with larger cohorts are warranted.

## Introduction

Diabetic retinopathy (DR) affects an estimated one in three people with diabetes mellitus (DM) (1) and causes severe visual impairment. Diabetic macular edema (DME), a common complication of DR, can present in both non-proliferative diabetic retinopathy (NPDR) and proliferative diabetic retinopathy (PDR) (2).

DME is pathologically linked to the disruption of the blood retinal barrier. In the hypoxic microenvironment of DR, vascular endothelial growth factor (VEGF) increases capillary permeability and breaks down blood retinal barrier (3). Ranibizumab (Lucentis, Genentech Inc., South San Francisco, CA), an anti-angiogenic agent, has revolutionized the treatment of DME. The RISE and RIDE phase II trial showed that 44.8% of patients gain more than 15 letters in vision after monthly injections of 0.3 mg ranibizumab (4). The success of ranibizumab over intravitreal steroid and photocoagulation monotherapy has also been established in the literatures (5, 6).

Nonetheless, monthly injections of ranibizumab is impractical as the cost of anti-VEGF agents and the requirement of frequent clinic visits be barriers to patient compliance to regimen (7). The TREX-DME study demonstrated that treat and extend (T&E) dosing was comparable with monthly dosing and allows for incremental increases in treatment intervals by 2 weeks. This resulted in less frequent injections and less expenditure (8). Therefore, T&E dosing with 4-week intervals may be more practical in terms of reducing treatment burden.

Despite robust findings from clinical trials, around half of eyes do not fully respond to anti-VEGF (9), and further exploration of prognostic factors associated with better visual outcomes is warranted. Age, HbA1c status, central retinal thickness (CRT) have been investigated but to mixed results (10–13). Moreover, little is known regarding how optical coherence tomography (OCT) biomarkers including epiretinal membrane (ERM) and ellipsoid zone disruption (EZD) affect the resolution of macular edema and final vision, and its implication for therapeutic strategy.

This tri-center 12 month study aims to investigate the efficacy of ranibizumab on DME following a regimen of 3 monthly loading injections plus 4-week T&E therapeutic intervals. To understand favorable factors for functional and anatomical visual outcomes, we assessed clinical parameters of patients with different therapeutic responses.

## Materials and Methods

### Subjects

This retrospective study was conducted from 2017 to 2019 at the Department of Ophthalmology of three tertiary centers in Taiwan (China Medical University Hospital, National Taiwan University Hospital, and Far Eastern Memorial Hospital). We reviewed subjects with either type I or type II DM and a concomitant DR diagnosis. DME diagnosis was made according to features of exudates and macular thickening on fundus and OCT exam. CRT was calculated as the average thickness of the central 1,000 μm diameter area (14) with spectral domain OCT (SD-OCT) device (Heidelberg Engineering, Heidelberg, Germany). Among the OCT biomarkers, EZD is defined as having any discontinuity of the second hyper-reflective layer of fovea on OCT. The shadowing effect of cysts and retinal vessels was not regarded as part of the EZD (15).

Inclusion criteria for receiving ranibizumab were as follows: eyes having Snellen best-corrected visual acuity (BCVA) between 20/400 and 20/40, CRT on OCT being >300 μm at the initial presentation, and eyes demonstrating late onset hyperfluorescence typical of macular leakage on fluorescence angiography (FA). Exclusion criteria involves having macular edema of non-diabetic causes, a history of vitrectomy and laser photocoagulation 3 months prior to study entry. Among PDR patients, subjects who were treated with additional laser photocoagulation during the study period were excluded. OCT images of poor quality were excluded as well.

The study protocol was approved by the Institutional Review Board and informed consent was obtained from all participants. The study complies with the tenets of the Declaration of Helsinki.

### Study Design and Statistical Analysis

**Figure 1:**
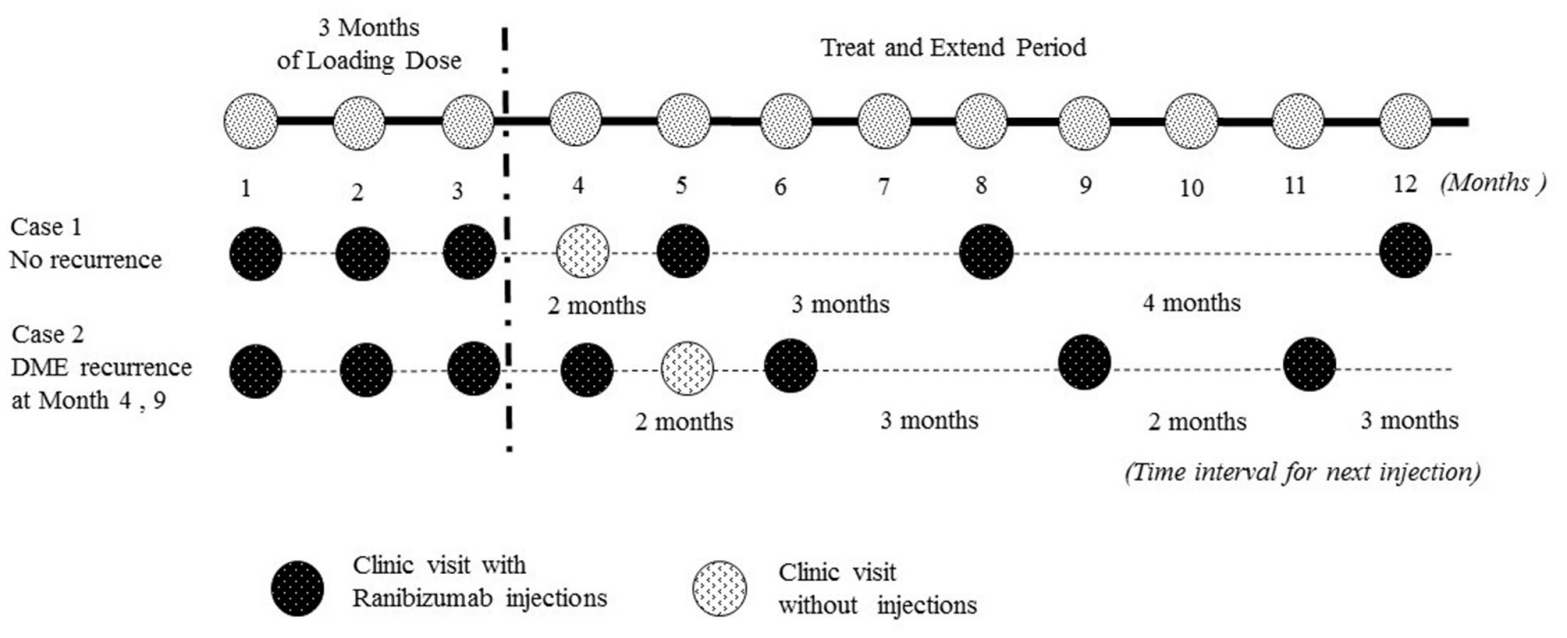
Illustrated the treat and extend (T&E) dosing algorithm in our real world study to determine treatment intervals. In this study, there were 3 monthly injections of ranibizumab, and injection intervals were extended by 4 weeks if there was no DME recurrence. DME recurrence was defined as having central retinal thickness >300 μm. If there was no recurrence, patients were allowed to extend their clinical visit and injection one more month due to personal reasons. BCVA, best-corrected visual acuity; DME, diabetic macular edema.

All patients received 3 monthly loading injections of 0.5 mg ranibizumab, followed by a T&E algorithm in which the treatment intervals were increased by 4 weeks after reaching a stable BCVA status and a CRT <300 μm. Ranibizumab injections interval were reduced by 4 weeks if the individual had vision loss due to DME recurrence. DME recurrence was defined as having CRT >300 μm. If there was no recurrence, patients were allowed to extend their clinical visit and injection one more month due to personal reasons (Figure 1). All subjects were followed for at least 12 months.

Primary outcomes included variations in BCVA and CRT after 12 months of treatment. Secondary outcomes were the univariate analysis and multivariate logistic regression analysis of the biomarkers that predicted better BCVA outcomes in DME. We applied Chi-square for the univariate analysis of categorical variable and ANOVA for numerical variable. In the multivariate logistic regression analysis, eventual CRT, BCVA changes and injection times were dependent variables. Baseline parameters such as age, DR staging and OCT biomarkers were independent variables. Statistical analysis was conducted with Statistical Package for the Social Sciences (SPSS) version 22.0 for Windows.

## Results

Ninety-one eyes of 64 patients with DME were enrolled. Thirty five (54.7%) males and 29 (45.3%) females were included. The baseline HbA1C was 7.44 ± 1.02 %. There were 4 eyes of mild NPDR, 14 of moderate NPDR, 28 of severe NPDR, 6 of PDR, and 39 of eyes with PDR that had received PRP (Table 1). The majority of DR staging was severe NPDR and PDR.

**Table 1:** Demographic data.

**Baseline characteristics**	**All patients (*N* = 64), Eyes (*n* = 91)**
Age	60.31 ± 10.75
Gender	
Female	29/64 (45.3%)
HbA1C (%)	7.44% ± 1.02%
CRT (μm)	411.30 ±114.61
LogMAR	0.58 ± 0.36
DR staging	
Mild NPDR	4/91 (4.40%)
Moderate NPDR	14/91 (15.4%)
Severe NPDR	28/91 (30.8%)
PDR	6/91 (6.59%)
PDR with PRP	39/91 (42.9%)
IRC	71/91 (78.0%)
HE	70/91 (76.9%)
DRIL	31/91 (34.1%)
EZD	26/91 (28.6%)
ERM	23/91 (25.3%)
SRF	18/91 (19.8%)

*N, number; CRT, Central Retinal Thickness; DR, Diabetic Retinopathy; NPDR, Non Proliferative Diabetic Retinopathy; PDR, Proliferative Diabetic Retinopathy; PRP, Panretinal Photocoagulation; IRC, Intra-Retinal Cyst; HE, Hard Exudate; DRIL, Disorganization of Retinal Inner Layers; EZD, Ellipsoid Zone Disruption; ERM, Epiretinal Membrane; SRF, Subretinal Fluid*.

**Figure 2:**
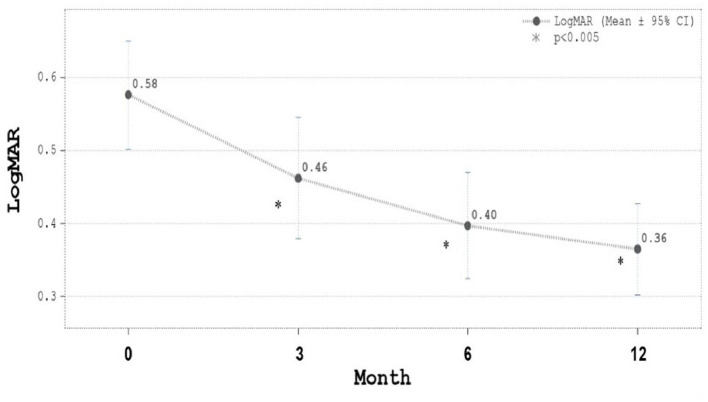
Best-corrected visual acuity (BCVA) in LogMAR during ranibizumab treatment. LogMAR was 0.58 ± 0.36 (mean ± SD) at baseline, 0.46 ± 0.40 μm at Month 3, 0.40 ± 0.35 at Month 6, and 0.36 ± 0.30 at Month 12. Mean LogMAR BCVA improved significantly from 0.58 at baseline to 0.36 in month 12.

**Figure 3:**
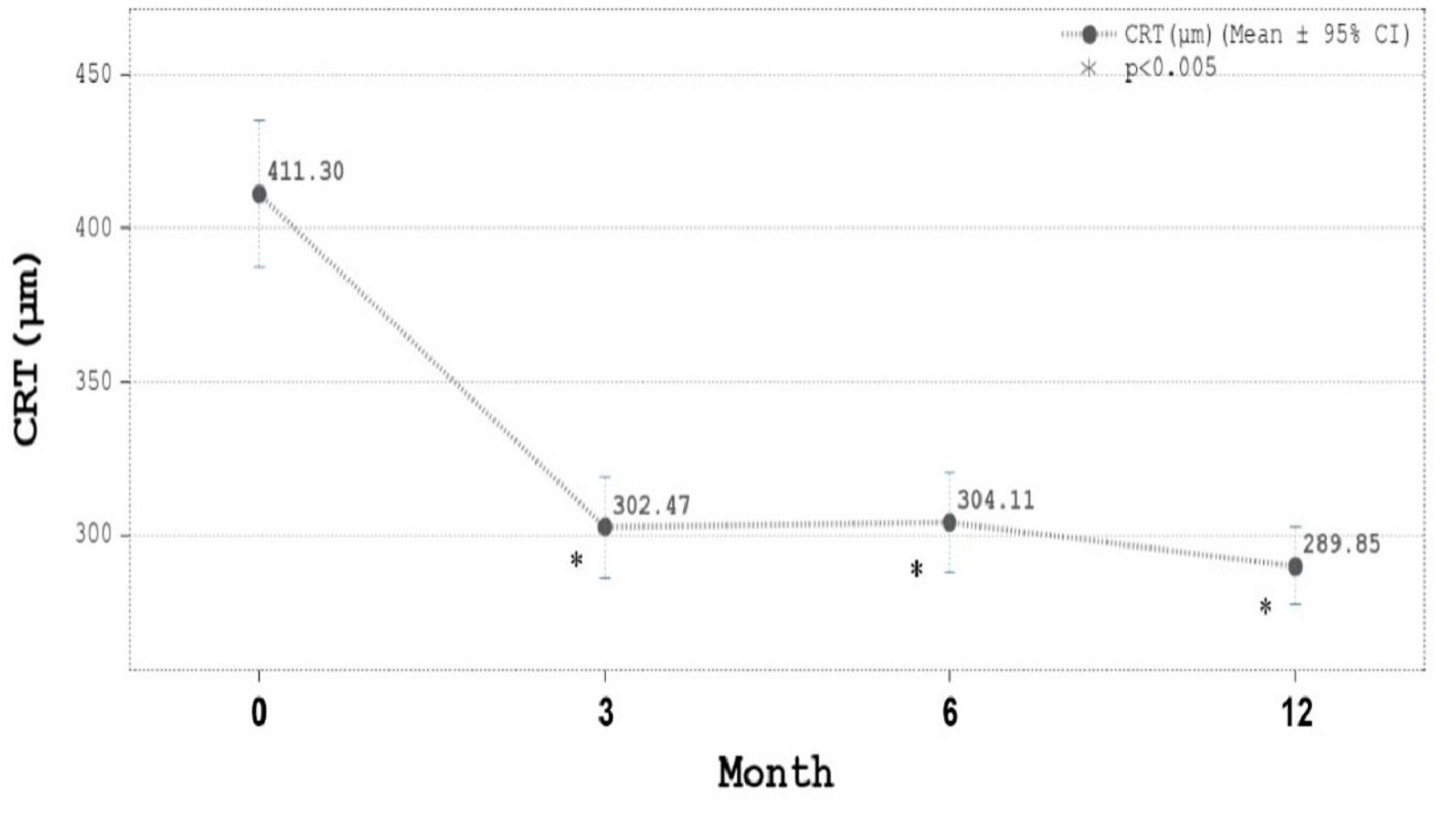
Central retinal thickness (CRT, μm) during ranibizumab treatment. CRT was 411.30 ± 114.61 μm (mean ± SD) at baseline, 302.47 ± 79.22 μm at Month 3, 304.11 ± 78.59 at Month 6, and 289.85 ± 60.71 μm at Month 12. Mean CRT decreased significantly from 411 μm at baseline to 289 μm in month 12.

The mean injection number was 7.67 ± 2.09 (5–12; 95% confidence interval) with 71.21% eyes received five to eight injections. The majority of cases needed eight injections. The proportion of eyes with BCVA improvement had gradually increased from 58.2% since Month three and reached 72.5% at month 12. The mean LogMAR BCVA improved significantly from 0.58 at baseline to 0.36 in month 12 (Figure 2). The mean CRT decreased significantly from 411.3 μm at baseline to 289.8 m in month 12 (Figure 3). In both Figures 2, 3, all the *p*-values shown were compared to the baseline LogMAR and baseline CRT, respectively.

Next, we performed inter-cohort univariate and multivariate analysis. Patients were further classified according to final CRT thickness (> or <300 μm), the final change in BCVA (with or without BCVA improvements) and total injection times (more or fewer than six injections) (Table 2). In the inter-cohort analysis of final CRT > or <300 μm, eyes of younger age and eyes having thinner baseline CRT, with EZD, and without ERM were associated with better final CRT (<300 μm) (Table 2). This correlation were also confirmed in multivariate analysis, where older age (odds ratio = 1.094, *p* = 0.0115), thicker CRT at study entry (odds ratio = 1.009, *p* = 0.0013), having ERM (odds ratio = 3.619, *p* = 0.0256) and without EZD (odds ratio = 0.127, *p* = 0.0045) were associated with worse final CRT (Table 3).

**Table 2:** Intercohort univariate analysis.

**Baseline**	**Grouped By final CRT**			**Grouped By final VA change**			**Grouped By injection times**		
**Clinical data**	**< 300 μm**	**≥ 300 μm**		**Improved**	**Not improved**		**≤ 6 shots**	**> 6 shots**	
Age (years)	**57.64** **±** **11.66**	**64.48** **±** **7.63**	*p* < 0.05^†^	60.85 ± 9.19	58.82 ± 14.45	NS	59.30 ± 13.14	61.05 ± 8.71	NS
Gender (Female)	20/39 (51.3%)	9/25 (36.0%)	NS	21/47 (44.7%)	8/17 (47.1%)	NS	12/27 (44.4%)	17/37 (45.9%)	NS
HbA1c (%)	7.47 ± 1.03	7.40 ± 1.02	NS	7.47 ± 0.92	7.37 ±1.29	NS	7.24 ± 0.98	7.59 ± 1.04	NS
CRT (μm)	**392.76** **±** **108.58**	**445.47** **±** **119.24**	*p* < 0.05^†^	421.36 ± 118.39	384.72 ± 101.42	NS	**366.58** **±** **86.84**	**434.40** **±** **120.87**	*p* < 0.05^†^
LogMAR	0.59 ± 0.37	0.55 ± 0.33	NS	**0.63** **±** **0.36**	**0.42** **±** **0.29**	*p* < 0.05	**0.47** **±** **0.30**	**0.63** **±** **0.37**	*p* < 0.05^†^
DR staging/OCT Biomarkers									
Mild NPDR	3/59 (5.08%)	1/32 (3.13%)	NS	**1/66 (1.52%)**	**3/25 (12.0%)**	*p* < 0.05	3/31 (9.68%)	1/60 (1.67%)	NS
Moderate NPDR	9/59 (15.3%)	5/32 (15.6%)	NS	11/66 (16.7%)	3/25 (12.0%)	NS	5/31 (16.1%)	9/60 (15.0%)	NS
Severe NPDR	16/59 (27.1%)	12/32 (37.5%)	NS	**25/66 (37.9%)**	**3/25 (12.0%)**	*p* < 0.05	7/31 (22.6%)	21/60 (35.0%)	NS
PDR	3/59 (5.08%)	3/32 (9.38%)	NS	4/66 (6.06%)	2/25 (8.00%)	NS	2/31 (6.45%)	4/60 (6.67%)	NS
PDR s/p PRP	28/59 (47.5%)	11/32 (34.4%)	NS	25/66 (37.9%)	14/25 (56.0%)	NS	14/31 (45.2%)	25/60 (41.7%)	NS
SRF (+)	14/59 (23.7%)	4/32 (12.5%)	NS	13/66 (19.7%)	5/25 (20.0%)	NS	4/31 (12.9%)	14/60 (23.3%)	NS
IRC (+)	45/59 (76.3%)	26/32 (81.3%)	NS	54/66 (81.8%)	17/25 (68.0%)	NS	25/31 (80.6%)	46/60 (76.7%)	NS
ERM (+)	**10/59 (16.9%)**	**13/32 (40.6%)**	*p* < 0.05^†^	**21/66 (31.8%)**	**2/25 (8.00%)**	*p* < 0.05^†^	5/31 (16.1%)	18/60 (30.0%)	NS
EZD (+)	**21/59 (35.6%)**	**5/32 (15.6%)**	*p* < 0.05^†^	**24/66 (36.4%)**	**2/25 (8.00%)**	*p* < 0.05^†^	7/31 (22.6%)	19/60 (31.7%)	NS
DRIL (+)	20/59 (33.9%)	11/32 (34.4%)	NS	23/66 (34.8%)	8/25 (32.0%)	NS	9/31 (29.0%)	22/60 (36.7%)	NS
HE (+)	45/59 (76.3%)	25/32 (78.1%)	NS	53/66 (80.3%)	17/25 (68.0%)	NS	23/31 (74.2%)	47/60 (78.3%)	NS

*CRT, Central Retinal Thickness; DR, Diabetic Retinopathy; NPDR, Non Proliferative Diabetic Retinopathy; PDR, Proliferative Diabetic Retinopathy; PRP, Panretinal Photocoagulation; SRF, Subretinal Fluid; IRC, Intra-Retinal Cyst; ERM, Epiretinal Membrane; EZD, Ellipsoid Zone Disruption; DRIL, Disorganization of Retinal Inner Layers; HE, Hard Exudate. N: Indicates a non-statistically significant*.

† *Indicates that statistical significance in this univariate analysis was also confirmed in multivariate analysis in Table 3. The bold values indicate p < 0.05*.

**Table 3:** Multiple logistic regression.

**Parameter**	**DF**	**Estimate**	**Standard Error**	**Wald Chi-Square**	***P*-value**	**Odds ratio**	**95% Wald**
**Dependent variable: Final CRT** **≥** **300** **μm (event) vs. Final CRT** **<** **300** **μm**							
Intercept	1	−10.0342	2.8891	12.0624	0.0005		
Age	1	0.0897	0.0355	6.3803	0.0115	1.094	1.020–1.173
ERM (+)	1	0.6431	0.2882	4.9807	0.0256	3.619	1.170–11.20
EZD (+)	1	−1.0323	0.3638	8.0525	0.0045	0.127	0.030–0.528
Initial CRT	1	0.00873	0.00272	10.2745	0.0013	1.009	1.003–1.014
**Dependent variable: Final VA not improved (event**, **ΔLogMAR** **≥** **0) vs. Final VA improved**							
Intercept	1	−2.1631	0.5133	17.7602	<.0001		
ERM (+)	1	−0.9148	0.3991	5.2546	0.0219	0.160	0.034–0.767
EZD (+)	1	−1.0054	0.3960	6.4451	0.0111	0.134	0.028–0.632
**Dependent variable: Injections** **>** **6 shots (event) vs. Injection times** **≤** **6 shots**							
Intercept	1	−1.9051	0.9950	3.666	0.056		
Initial CRT	1	0.0065	0.0025	6.507	0.011	1.006	1.002–1.012

*Other Variables included before model selection were as following: gender, age, HbA1C, initial LogMAR, initial CRT, DR staging and initial OCT data. CRT, Central Retinal Thickness; DR, Diabetic Retinopathy; EZD, Ellipsoid Zone Disruption; ERM, Epiretinal Membrane*.

Comparing the two groups with or without BCVA improvements, we observed that patients having worse baseline BCVA, severe baseline NPDR status, and having ERM or EZD were associated with significant logMAR BCVA improvements in univariate analysis. Subjects with mild NPDR status were not associated with significant BCVA recovery (Table 2). However, in multivariate analysis, only having ERM (ERM with no final BCVA recovery, odds ratio = 0.160, *p* = 0.0219) or EZD (EZD with no final BCVA recovery, odds ratio = 0.134, *p* = 0.0111) was associated with significant final BCVA recovery.

Searching for factors predictive of frequent injections, we discovered that eyes with thicker CRT and worse BCVA at study entry were associated with more Ranibizumab injections in the univariate analysis (Table 2). Multivariate analysis confirmed that only thicker baseline CRT was predictive of receiving more than six injections (odd ratio = 1.006, *p* = 0.011) (Table 3).

Among the OCT biomarkers, only the presence of EZD and ERM significantly influenced the final BCVA improvements and final CRT (Tables 2, 3). No OCT biomarkers statistically significantly influenced the total injection times in both univariate and multivariate analysis (Tables 2, 3).

## Discussion

We have demonstrated the efficacy of T&E regimen with 0.5 mg ranibizumab by 4-week intervals. On average, the BCVA gain was 10 letters after 12 months. These results were concordant with the clinical trials that adopt extended 2-week treatment intervals. For instance, in the TREX-DME 1-year study, a standard interval of 2 weeks was adopted and BCVA gains were 9.6 and 9.5 letters for the respective T&E and T&E plus laser arms (16). Our study, though adopting an interval twice as long as the standard interval, attained similar BCVA gains as in the TREX-DME 1-year study. Moreover, on average, only eight injections per year were required to reach efficacy, whereas the TREX-DME 1-year study required 10.7 injections (16). Hence, we demonstrated that a longer 4-week T&E interval is feasible and economical for achieving adequate control of DME.

In the present analysis, ranibizumab reduced the average CRT by 121 μm at month 12 (Figure 3). Similarly, in the TREX-DME trial, CRT was reduced by 123 and 146 μm, respectively, for monthly and T&E regimens. No statistical significance were observed between the two protocols in that trial (8). Interestingly, we demonstrated that the improvements of BCVA and CRT were parallel and significant starting from month 3 and until month 12 (Figures 2, 3). Moreover, the greatest increases in both BCVA and CRT were observed after 3 monthly loading injections. Protocol I from Diabetic Retinopathy Clinical Research (DRCR) and research by Lai et al. had similar results in demonstrating that BCVA restoration after the 3 monthly injections was predictive of long-term visual benefits (12, 17).

Even though we have established the efficacy of ranibizumab in DME as in other clinical trials, 35.6 and 27.5% of our patients, respectively, failed to response in CRT and BCVA under the study protocol. In both univariate and multivariate analysis, we discovered that younger age, initial CRT, and the presence of ERM or EZD were significant clinical parameters that influenced final CRT outcomes. Younger ages were, in previous studies, correlated with better vision recovery but its relation to final CRT outcomes had not been established (10, 18). However, the age was not significantly associated with BCVA recovery in our study. Instead, we found that age was only predictive of thinner final CRT.

Aside from age, we found that thinner baseline CRT was predictive of better final CRT while thicker baseline CRT is predictive of worse final CRT outcome. Bressler et al. proposed that a thicker baseline CRT may result in a failure to achieve the ideal final CRT of <250 μm, but may still attain greater reduction of macular edema in the end (10). Hence, eyes having more severe DME with thicker CRT at the beginning may still benefit from anti-VEGF injections.

Whether baseline CRT predicts better BCVA recovery is controversial. While eyes with thinner retinal thickness is expected to have lesser capacity for BCVA improvements, baseline CRT in our study did not make a difference in final vision recovery. Contrary to our present analysis, the RESTORE trial reported that eyes with thicker initial CRT experienced greater VA gains (6). Of note, in this trial baseline BCVA of the thicker retina was not adjusted and may be a confounding factor to vision gain analysis. In later literature, Well et al. and others have demonstrated that baseline BCVA is a stronger predictor of visual improvement than retinal thickness (19, 20). Also, it has been observed that visual acuity may not be compatible with a given degree of macular edema. That is, one may have better gain in vision but develop a paradoxical increase in retinal thickness (21). Therefore, though it is possible that retinal thickness is modestly related to functional vision outcome, its impact may not be as essential as initial BCVA status.

Since factors other than CRT reductions relate to vision improvement, researchers have explored the microstructure of the retina in search of other co-variables (13, 22). The presence of photoreceptor integrity and the co-existence of vitreoretinal interface (VRI) abnormality may affect visual outcome (23). In our study, eyes with EZD had thinner final CRT compared with eyes without EZD. We hypothesized that the reduction of CRT is related to external limiting membrane (ELM) defect. ELM is considered as the organized layer that comprised of the cellular attachment between Muller glial cells and contact between Muller cells and photoreceptors (24, 25). In addition to the anatomical location of the ELM, the presence of tight junction proteins such as occludins on it, further support the notion that, the ELM serves as a retinal barrier between the inner retinal layer and outer photoreceptor segments. In eyes with DME, there are ELM defect due to swollen Muller glial cells and further loss of occludin proteins (24, 25). Though our study did not measure the continuity of the ELM on OCT, ELM disruption has frequently been associated with EZD (26). Under this premise, final CRT reduction in eyes with EZD may be related to ELM defects, which facilitate the pumping function of RPE and lead to the reduction of intra-retinal fluid. Hence, under the effect of ranibizumab, eyes with EZD would have better CRT outcome than those without EZD.

One may propose that CRT reductions in EZD are related to fovea atrophy instead of true therapeutic effects seen in DME patients. However, in reviewing our data, we found that the final average CRT thickness in EZD group was 289.85 ± 60.71 μm at 1 year, and only one eye with EZD had a CRT thickness of <200 μm. Our CRT data of EZD group was far thicker than the criteria set for fovea atrophy, which was as either <200 or 150 μm (27, 28). Hence, fovea atrophy was unlikely the contributing factor in our study. We suppose it was the therapeutic effect of ranibizumab and ELM disruption that led to thinner final CRT in the EZD group.

Vitreoretinal interface abnormality encompasses disorders such as ERM, vitreomacular adhesion (VMA), and vitreomacular traction (VMT), and affects the vision outcome of DME patients. Kulikov et al. revealed that the presence of ERM, VMT, and VMA were associated with less CRT reduction after anti-VEGF injections. However, this study did not analyze BCVA gains and was involved a 1 month period, long term outcomes uncertain (29). Others have found that anti-VEGF is still beneficial for eyes with VMA (30), while some have observed no difference in vision recovery and CRT reduction in VMA and ERM group (13, 31). VMT was, however, mostly indicative of poor visual outcome (29, 31). The traction from partial posterior vitreous detachment in VMT might lead to the distortion of inner retinal layer and thus adverse outcomes.

On the other hand, whether ERM precludes better BCVA gain is controversial. Intriguingly, we found that eyes with ERM have greater BCVA improvements than eyes without ERM. Other parameters such as initial CRT or the presence of DRIL, IRC, and SRF did not affect BCVA improvement. Similar to our observation, other studies had also found better vision recovery in eyes with ERM under anti-VEGF therapy; however, the mechanism is not well-known (32). Some proposed that in the absence of VMT and fibrovascular proliferation, ERM did not contribute to the difference to final BCVA (12). From our data, we conclude that the presence of ERM leads to at least non-inferior BCVA recovery. It is difficult to ascertain whether having ERM lead to superior visual outcome, so further study with larger cohort is warranted. Therefore, in our study the presence of ERM should not preclude treatment with anti-VEGF since the presence of ERM does not hinder BCVA improvements.

Our study is limited as it is retrospective in nature. We acknowledge that there are no matching controls and confounding factors may exist in inter-cohort analysis. In inter-cohort analysis, parameters such as age, gender, and HbA1c were analyzed by individuals. For DR staging and OCT parameters, the data were analyzed by eyes. This difference did not influence the univariate analysis, but may lead to some bias in multivariable analysis. Analyzing only one eye from one person will resolve this problem but will also decrease the available data. For the current study, consideration of sample size still outweighs the existence of possible bias. Second, this study is of a relatively small sample size with short follow-up period. This might affect the statistical power in regression model analysis. However, most factors identified as significant in univariate analysis were also confirmed in multivariate regression.

Notwithstanding its limitations, our study bears significance in identifying that the presence of ERM should not preclude the individual from receiving anti-VEGF therapy. Additionally, we demonstrate how ranibizumab therapy affects retinal microstructure both anatomically and functionally.

In conclusion, the T&E regimen in a 4-week interval with ranibizumab was a feasible and economical option for patients with DME. Parameters including age, initial CRT, and presence of ERM significantly influenced the outcome of T&E regimen. Moreover, the presence ERM should not preclude patients from receiving anti-VEGF therapy. Further study with larger cohorts is warranted.

## Data Availability Statement

The raw data supporting the conclusions of this article will be made available by the authors, without undue reservation.

## Ethics Statement

The studies involving human participants were reviewed and approved by China Medical University Hospital Institutional Review Board. The patients/participants provided their written informed consent to participate in this study.

## Author Contributions

C-JL, C-TL, Y-TH, and J-KW contributes to conception and design of the study. C-JL, C-TL, Y-TH, J-KW, N-YH, H-SC, C-YC, and S-WW participated in data acquisition. C-JL, C-TL, C-YL, HB, N-YH, H-SC, C-YC, and S-WW analyzed and interpreted the data set. C-JL, C-TL, C-YL, HB, and N-YH drafted the manuscript. C-JL and C-TL supervised and revised the manuscript to meet academic standards. All authors had approved the integrity of the manuscript.

## Conflict of Interest

The authors declare that the research was conducted in the absence of any commercial or financial relationships that could be construed as a potential conflict of interest.
